# Estimation of cardiac output and peripheral resistance using square-wave-approximated aortic flow signal

**DOI:** 10.3389/fphys.2012.00298

**Published:** 2012-07-25

**Authors:** Nima Fazeli, Jin-Oh Hahn

**Affiliations:** Department of Mechanical Engineering, University of Alberta, EdmontonAB, Canada

**Keywords:** cardiovascular system, cardiac output, peripheral resistance, windkessel model, pressure-dependent arterial compliance

## Abstract

This paper presents a model-based approach to estimation of cardiac output (CO) and total peripheral resistance (TPR). In the proposed approach, the response of cardiovascular system (CVS), described by the windkessel model, is tuned to the measurements of systolic, diastolic and mean arterial blood pressures (BP) so as to yield optimal individual- and time-specific system time constant that is used to estimate CO and TPR. Unique aspects of the proposed approach are that it approximates the aortic flow as a train of square waves and that it also assumes pressure-dependent arterial compliance, as opposed to the traditional windkessel model in which aortic flow is approximated as a train of impulses and constant arterial compliance is assumed. It was shown that the proposed model encompasses the standard windkessel model as a limiting case, and that it also yields more realistic BP waveform response than the standard windkessel model. The proposed approach has potential to outperform its standard counterpart by treating systolic, diastolic, and mean BP as independent features in estimating CO and TPR, rather than solely resorting to pulse pressure as in the case of the standard windkessel model. Experimental results from *in-vivo* data collected from a number of animal subjects supports the viability of the proposed approach in that it could achieve approximately 29% and 24% reduction in CO and TPR errors when compared with its standard counterpart.

## Introduction

Cardiac output (CO) is one of the most important hemodynamic parameters to be monitored and assessed in ambulatory and critically ill patients (Jansen et al., 1990). It is frequently used for disease diagnostics and monitoring (Heldt, 2006). It is also a very important hemodynamic variable for therapeutic titrations (Heldt, 2006). In contrast to the use of arterial blood pressures (BP) [which is a late indicator of hemodynamic instability (Barcroft et al., 1944)], CO allows early detection of hemodynamic collapse. Despite its clinical significance, direct measurement of CO is extremely difficult. Presently, the clinical gold standard accepted for CO measurement is the thermo-dilution (Ganz et al., 1971), but it is known to be a highly invasive procedure that has limited accuracy (Botero et al., 2004) and may incur cardiovascular risk (Manecke et al., 2002). Non-intrusive techniques such as echo-cardiography (Ihlen et al., 1984) and electrical velocimetry (Suttner et al., 2006; Zoremba et al., 2007) are promising alternatives, but often their accuracy is not satisfactory enough yet to be clinically applicable (Siegel and Pearl, 1992).

In order to overcome these drawbacks, efforts have been made to estimate CO from arterial BP waveform(s) (see Liljestrand and Zander, 1928; Welkowitz et al., 1991; Martin et al., 1994; Redling and Akay, 1997; Jansen et al., 2001; Ishihara et al., 2004; Mukkamala et al., 2006; Parlikar et al., 2007; Xu et al., 2009; Arai et al., 2010; Reisner et al., 2011 for examples of recent efforts), which are collectively known as the pulse contour methods. In this framework, CO is estimated using the morphological features in the BP waveform(s). Most of the existing pulse contour methods are built upon the windkessel model of cardiovascular system (CVS) that involves lumped arterial compliance and total peripheral resistance (TPR) [e.g., Modelflow (Jansen et al., 2001; Reisner et al., 2011) and pulse pressure methods (Reisner et al., 2011) and its variants (Ishihara et al., 2004), cycle-averaged windkessel model-based method (Jansen et al., 2001), hybrid windkessel model-based method (Jansen et al., 1990)], although there are methods based on empiric features in the arterial BP waveforms (Liljestrand and Zander, 1928; Parlikar et al., 2007; Arai et al., 2010), more detailed distributed-parameter models (Martin et al., 1994; Redling and Akay, 1997) and black-box models combined with advanced signal processing (Welkowitz et al., 1991; Mukkamala et al., 2006; Xu et al., 2009).

Inspired by its wide acceptance and frequent application in CO and TPR estimation, this study aims at developing a universal approach that has potential to enhance the efficacy of CO and TPR estimation based on the standard windkessel model (Frank, 1930) (collectively referred to as the standard windkessel-model-based method hereafter). In this method, the aortic flow signal is approximated as an impulse train, which essentially yields a CO estimator based on the pulse pressure (see Section “Methods” for details). Noting that a number of existing developments on CO estimation (Jansen et al., 2001; Ishihara et al., 2004; Parlikar et al., 2007; Reisner et al., 2011) are variants and/or extensions of this traditional method, it is anticipated that successful improvement of CO and TPR estimation methods based on the standard windkessel model with aortic flow approximated as impulse train has potential to enhance its variants and/or extensions as well. In this study, we focus on two main opportunities to enhance the CO and TPR estimation efficacy of the standard windkessel-model-based method: (1) to use a better approximation of aortic flow signal that can result in more realistic BP waveform(s), and (2) to exploit independent morphological features in the arterial BP waveform more rigorously rather than solely relying on the pulse pressure as in the standard CO estimator implemented with traditional windkessel model.

This paper presents a new universal approach to the estimation of CO and TPR that can improve the efficacy of windkessel model-based CO and TPR estimation methods. In this approach, the CVS model is characterized using the measurements of systolic, diastolic, and mean BP. In contrast to the standard windkessel-model-based approach to CO and TPR estimation (which approximates the aortic flow signal as a train of impulses), the proposed approach uses the aortic flow signal that is approximated as a train of square waves, which can yield morphologically more realistic arterial BP waveforms. It is shown that the proposed method encompasses its standard counterpart as a limiting case. It is also suggested that the proposed method can outperform the standard method by treating systolic, diastolic, and mean BP as independent features, rather than solely resorting to a single feature (i.e., the pulse pressure) as in the standard method. Experimental results from *in-vivo* animal study illustrated that the proposed approach could achieve 29% and 24% reduction in CO and TPR errors against the standard windkessel model-based method. We anticipate that the proposed approach can be combined with a variety of existing windkessel model-based CO and TPR estimation methods to enhance the methods, accuracy, and reliability.

This paper is organized as follows. Section “Methods” describes the proposed approach to CO and TPR estimation by comparing it to the standard windkessel-model-based method. Section “Methods” presents the details of experimental protocol and data analysis. Section “Results” presents the results, which are discussed in Section “Discussion”. Section “Conclusion” concludes the paper with future directions.

## Methods

**Figure 1:**
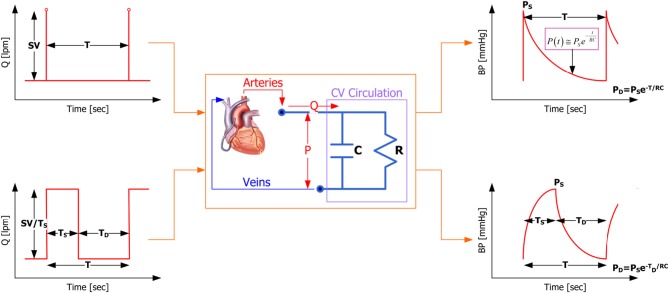
**Standard versus proposed windkessel-model-based CVS model characterization methods**.

Our proposed method is built upon the two-parameter windkessel model of the CVS (see Figure 1), which is essentially an electrical circuit consisting of a capacitor (to represent the compliance of the conduit arteries) and a resistor (to represent the resistance of the peripheral arteries). First of all, the governing equation for the two-parameter windkessel model is given by (1):

(1) $$\frac{dP}{dt}=-\frac{1}{RC}P+\frac{1}{C}Q$$

where *P* is BP and *Q* is aortic flow. Figure 1 demonstrates that the proposed windkessel method has strengths over its standard counterpart in that (1) by using the aortic flow signal approximated as a train of square waves, the resulting BP waveform is morphologically more realistic compared with its standard counterpart (see the upper and lower right corners of Figure 1 for BP waveforms resulting from impulse and square wave trains, respectively), and (2) it exploits the features in BP waveform more rigorously, i.e., it uses systolic, diastolic, and mean BP as independent features to characterize the CVS model and estimate CO and TPR, whereas the standard method depends solely on the pulse pressure.

To clearly demonstrate the distinction between the standard and the proposed estimation methods for CO and TPR, the standard windkessel method is first introduced, and then the proposed windkessel method is described in detail.

### Standard windkessel method

Given the aortic flow signal approximated as a train of impulses shown in the upper left corner of Figure 1, which has amplitude equal to the stroke volume (SV) and the period equal to a cardiac cycle (i.e., heart period) *T*, the resulting BP response of the windkessel model (1) is given by:

(2) $$P(t)=P_{D}e^{-\frac{t}{RC}}+\frac{\bar{Q}}{C}Te^{-\frac{t}{RC}},$$

where *P*_*D*_ is diastolic BP and $\bar{Q}$ is CO. Equation (2) is valid for a single cardiac cycle, i.e., 0 ≤ *t* ≤ *T*. The systolic as well as diastolic BP corresponds to the value of BP at *t* = 0 and *t* = *T*:

(3a) $$P_{S}=P_{D}+\frac{\bar{Q}}{C}T$$

(3b) $$P_{D}=P_{S}e^{-\frac{t}{RC}}$$

where *P*_*S*_ is systolic BP. Equation (3a) is of particular interest in estimating CO and TPR; rearranging it yields (4):

(4a) $$\hat{\bar{Q}}=C\frac{P_{S}-P_{D}}{T}\propto \frac{P_{P}}{T},$$

(4b) $$\hat{R}=\frac{1}{C}\bar{P}\frac{T}{P_{p}}\propto \bar{P}\frac{T}{P_{p}},$$

where $\hat{\bar{Q}}$ and $\hat{R}$ are the estimated CO and TPR, and *P*_*P*_ denotes the pulse pressure. Under the assumption that arterial compliance *C* remains constant over a time window under consideration, Equation (4) can be utilized to estimate the trend of CO and TPR as the ratio of pulse pressure and heart period as well as mean BP. Equation (4) can also be used to estimate the absolute CO and TPR if initial measurements to calibrate *C* are available. It is obvious that the only information the standard windkessel method exploits in the BP waveform for estimating CO and TPR is pulse pressure.

### Proposed windkessel method

In contrast to its standard counterpart, the proposed windkessel method for estimating CO and TPR intends to rigorously exploit multiple independent features in the BP waveform in characterizing the CVS model. For this purpose, the aortic flow signal is approximated as a train of square waves shown in the lower left corner of Figure 1, as opposed to a train of impulses used in the standard windkessel method (shown in the upper left corner of Figure 1). In this approximation, the amplitude of each square wave is given by $\bar{Q}\frac{T}{T_{S}}$ where *T*_*S*_ is the left ventricular ejection period, since the area under each square wave must be equal to SV. It then follows that solving Equation (1) for BP during 0 ≤ *t* ≤ *T*_*S*_ yields

(5) $$\begin{array}{c} P(t)=P_{D}e^{-\frac{t}{RC}}+\int_{0}^{t}e^{-\frac{t-\tau }{RC}}\frac{\bar{Q}}{C}\frac{T}{T_{S}}d\tau \\ =P_{D}e^{-\frac{t}{RC}}+R\bar{Q}\frac{T}{T_{S}}\left[1-e^{-\frac{t}{RC}}\right]\\ =P_{D}e^{-\frac{t}{RC}}+\bar{P}\frac{T}{T_{S}}\left[1-e^{-\frac{t}{RC}}\right]. \end{array}$$

During *T*_*S*_ ≤ *t* + *T*_*S*_ ≤ *T*, on the other hand, BP is given by

(6) $$\begin{array}{c} P(t)=P_{S}e^{-\frac{t}{RC}}\\ =P_{D}e^{-\frac{t+T_{S}}{RC}}+\bar{P}\frac{T}{T_{S}}e^{-\frac{t}{RC}}\left[1-e^{-\frac{T_{S}}{RC}}\right]. \end{array}$$

Based on (5) and (6), the following expressions for systolic and diastolic BP are obtained:

(7a) $$P_{S}=P_{D}e^{-\frac{T_{S}}{RC}}+\bar{P}\frac{T}{T_{S}}\left[1-e^{-\frac{T_{S}}{RC}}\right],$$

(7b) $$P_{D}=P_{S}e^{-\frac{T_{D}}{RC}}=P_{D}e^{-\frac{T}{RC}}+\bar{P}\frac{T}{T_{S}}e^{-\frac{T_{D}}{RC}}\left[1-e^{-\frac{T_{S}}{RC}}\right].\;$$

In addition, mean BP can be obtained as follows based on (5) and (6):

(8) $$\bar{P}=\frac{1}{T}\{\int_{0}^{T_{S}}P_{D}e^{-\frac{\tau }{RC}}+\bar{P}\frac{T}{T_{S}}\left[1-e^{-\frac{\tau }{RC}}\right]d\tau +\int_{0}^{T_{D}}P_{S}e^{-\frac{\tau }{RC}}d\tau .$$

In the proposed windkessel method, the model-based expressions in Equations (7) and (8) for systolic, diastolic, and mean BP are compared with the actual BP measurements, and the set of parameters in the windkessel model characterizing Equations (7) and (8), i.e., *RC*, *T*_*S*_ and *T*_*D*_, is optimized so that the discrepancy between the model-predicted versus actual systolic, diastolic, and mean BP are minimized. Specifically, the model-predicted systolic, diastolic and mean BP values are evaluated as follows:

(9a) $${\hat{P}}_{S}=P_{D}e^{-\frac{T_{S}}{RC}}+\bar{P}\frac{T}{T_{S}}\left[1-e^{-\frac{T_{S}}{RC}}\right],$$

(9b) $${\hat{P}}_{D}=P_{D}e^{-\frac{T}{RC}}+\bar{P}\frac{T}{T_{S}}e^{-\frac{T_{D}}{RC}}\left[1-e^{-\frac{T_{S}}{RC}}\right],$$

(9c) $$\begin{array}{c} \bar{P}=\frac{1}{T}\{\int_{0}^{T_{S}}P_{D}e^{-\frac{\tau }{RC}}+\bar{P}\frac{T}{T_{S}}\left[1-e^{-\frac{\tau }{RC}}\right]d\tau \\ \;+\int_{0}^{T_{D}}{\hat{P}}_{S}e^{-\frac{\tau }{RC}}d\tau \}, \end{array}$$

Noting that *T*_*S*_ + *T*_*D*_ = *T*, the optimal set of the windkessel model parameters {RC^*^, T^*^_S_} is determined by solving the following constrained optimization problem:s

(10a) $$\{RC^{*},T_{S}^{*}\}=\arg\min[\left\|P_{S}-{\hat{P}}_{S}\right\|+\left\|P_{D}-{\hat{P}}_{D}\right\|+\left\|\bar{P}-\hat{\bar{P}}\right\|],$$

where, with *T* inferred directly from measured BP, T^*^_D_ given by

(10b) $$T_{D}^{*}=T-T_{S}^{*}.$$

Using the CVS model parameters in (10a) thus identified, the trend of CO and TPR can be estimated in two alternative ways, depending on the assumption made in regards to the behavior of arterial compliance *C*: (1) constant or (2) pressure-dependent. First, assuming *C* is constant, the trend of CO can be estimated by dividing the measured mean BP by RC^*^, and the trend of TPR can be estimated directly by RC^*^:

(11a) $$\hat{\bar{Q}}\propto \frac{\bar{P}}{RC^{*}},$$

(11b) $$\hat{R}\propto RC^{*},$$

where $\hat{R}$ is the estimated TPR. On the other hand, if *C* is assumed to be pressure-dependent, its effect must be cancelled out in estimating the trend of CO and TPR. This can be accomplished by first dividing the measured mean BP by RC^*^, then multiplying *C* as a function of BP:

(12a) $$\bar{Q}=\frac{\bar{P}}{RC^{*}}\times C(P_{S},P_{D},\bar{P}),$$

(12b) $$\hat{R}=\frac{RC^{*}}{C(P_{S},P_{D},\bar{P})}.$$

Note that the relationships in Equation (12) are strict equalities. In this preliminary study, *C* is assumed as a simple monotonic linear function of mean BP, i.e.,

(13) $$C(P_{S},P_{D},\bar{P})={\text{η}}_{1}\bar{P}+{\text{η}}_{2}.$$

Using Equation (13), Equation (12) can be re-formulated into the following:

(14a) $$\hat{\bar{Q}}=\frac{\bar{P}}{RC^{*}}\times ({\text{η}}_{1}\bar{P}+{\text{η}}_{2})={\text{η}}_{1}\frac{{\bar{P}}^{2}}{RC^{*}}+{\text{η}}_{2}\frac{\bar{P}}{RC^{*}},$$

(14b) $$\hat{R}=\frac{RC^{*}}{C(P_{S},P_{D},\bar{P})}=\frac{RC^{*}}{{\text{η}}_{1}\bar{P}+{\text{η}}_{2}}.$$

One advantage of Equation (14) compared with Equation (11) is that it can accommodate into CO and TPR estimation the physiological nature of the arterial compliance that indeed changes with BP. However, a pre-calibration procedure is usually required to determine η_1_ and η_2_, since arterial compliance is rarely known a priori.

In essence, the proposed method is distinct from its standard counterpart in the sense that it regards systolic, diastolic and mean BP as independent features in characterizing the windkessel CVS model and estimating CO and TPR, whereas the standard method only concerns the pulse pressure.

## Methods

### Experimental protocol

Under the experimental protocol #01–055 approved by the MIT Committee of Animal Care, aortic flow and radial BP data were collected from eight anaesthetized swine subjects.

The chest was opened with a midline sternotomy. An ultrasonic flow probe was placed around the aortic root for the central aortic flow (T206 with A-series probes, Transonic Systems, Ithaca, NY). Besides, a 25-gauge angiocatheter was placed in the foreleg, distal to the brachial artery, and attached to an external pressure transducer via short, rigid tubing for the radial arterial BP. Each transducer output was interfaced to a microcomputer via an A/D conversion system (MP150WSW, Biopac Systems).

The physiological conditions of the swine subjects were widely altered in order to investigate how the CO estimation method behaves over a broad range of physiological conditions. The following interventions were performed to vary the physiological conditions of the swine subjects: the infusions of crystalloid volume, phenylephrine, dobutamine, isoproterenol, esmolol, nitroglycerine, and a progressive hemorrhage. The administration of each medicine was followed by a brief recovery period.

### Data collection, signal processing, and statistical analysis

The aortic flow and radial BP waveforms were first measured at 250 Hz without filtering from each swine subject, which were then pre-filtered using an FIR low-pass digital filter with 30 Hz cut-off frequency and down-sampled to 125 Hz. Following filtering and down-sampling, the aortic flow-radial BP data pair was segmented into 8 s-long time series sequences having 1000 data samples. Totally 4638 data segments were used in this study.

For each of the 8 s-long data segments obtained, CO and mean BP representative of a data segment were calculated by averaging the aortic flow and radial BP waveforms in the segment, respectively. The systolic and diastolic BP were calculated by averaging systolic and diastolic BP in all the cardiac cycles in the segment. In each data segment, the estimate of the trend of CO associated with the standard windkessel method was calculated based on the pulse pressure associated with the data segment using Equation (4). Also, the trend of TPR was obtained directly from dividing mean BP by the estimated trend of CO; $\hat{R}=\bar{P}\frac{T}{P_{P}}$. For the proposed method, the optimal windkessel model parameters {RC^*^, T^*^_S_, T*_D_} were determined by solving the constrained optimization problem in Equation (10) using the measurements of systolic, diastolic and mean BP associated with the data segment. Then the estimates of the trends of CO and TPR were calculated with Equations (11) and (14) for constant and pressure-dependent arterial compliance, respectively.

Once the measured versus estimated CO $\left\{{\bar{Q}}_{i},\;{\hat{\bar{Q}}}_{i}\right\}$ and TPR $\left\{R_{i},\;{\hat{R}}_{i}\right\}$ pairs for all the data segments were obtained for each swine subject (*i* = 1, … 8), the estimated CO and TPR were calibrated to the measurements via linear regression analysis in order to compare the estimates with the gold standard measurements. Specifically, the following calibration was applied to the standard windkessel method:

(15a) $$\bar{Q}=a_{1,Q}\hat{\bar{Q}}+a_{2,Q}=a_{1,Q}\frac{P_{P}}{T}+a_{2,Q},$$

(15b) $$R=a_{1,R}\hat{R}+a_{2,R}.$$

For the proposed windkessel method, different calibration procedure was applied to Equations (11) and (14). For CO and TPR estimation with constant arterial compliance, i.e., Equation (11), the calibration similar to Equation (15) was applied:

(16a) $$\bar{Q}=b_{1,Q}\hat{\bar{Q}}+b_{2,Q}=b_{1,Q}\frac{\bar{P}}{RC^{*}}+b_{2,Q},$$

(16b) $$R=b_{1,R}\hat{R}+b_{2,R}=b_{1,R}RC^{*}+b_{2,R}.$$

For CO and TPR estimation with pressure-dependent arterial compliance i.e., Equation (14), on the other hand, the slope of the linear regression must be unity because the relationship in Equation (14) are strict equalities. Therefore, a simple intercept calibration was applied to CO:

(17a) $$\bar{Q}=\hat{\bar{Q}}+{\text{η}}_{3}={\text{η}}_{1}\frac{{\bar{P}}^{2}}{RC^{*}}+{\text{η}}_{2}\frac{\bar{P}}{RC^{*}}+{\text{η}}_{3},$$

where η_1_ and η_2_ represent pressure-dependent arterial compliance [see Equation (14)], whereas the intercept η_3_ is intended to compensate for the inaccuracy in approximating arterial compliance to a monotonic linear function of mean BP. Using η_1_ and η_2_ obtained above, TPR is calibrated as follows:

(17b) $$R=c_{1,R}\hat{R}+c_{2,R}=\frac{RC^{*}}{{\text{η}}_{1}\bar{P}+{\text{η}}_{2}}+c_{2,R},$$

where *c*_1,*R*_ = 1 was assumed because Equation (14b) is strict equality.

Once calibrated, the fidelity of the standard and proposed CO and TPR estimation methods were assessed quantitatively by calculating (1) the coefficient of determination (CoD; *r*^2^ value), (2) the limits of agreement (i.e., the Bland-Altman statistics) between measured versus estimated CO and TPR, and (3) the root-mean-squared normalized errors (RMNSE) between measured versus estimated CO and TPR, respectively. RMNSE was calculated first for each swine subject as follows:

(18a) $$e_{i,Q}=100\times \sqrt{\frac{1}{N_{i}}\sum_{k=1}^{N_{i}}{\left[\frac{{\bar{Q}}_{i}(k)-{\hat{\bar{Q}}}_{i}(k)}{{\bar{Q}}_{i}(k)}\right]}^{2}},$$

(18b) $$e_{i,R}=100\times \sqrt{\frac{1}{N_{i}}\sum_{k=1}^{N_{i}}{\left[\frac{R_{i}(k)-{\hat{R}}_{i}(k)}{R_{i}(k)}\right]}^{2}},$$

where *N*_*i*_ is the total number of data segments associated with the *i*^th^ swine subject, ${\bar{Q}}_{i}(k)$ and ${\hat{\bar{Q}}}_{i}(k)$ are measured versus estimated (and calibrated) CO for the *k*^th^ data segment of the *i*^th^ swine subject, and *R*_*i*_(*k*) and ${\hat{R}}_{i}(k)$ are measured versus estimated (and calibrated) TPR for the *k*^th^ data segment of the *i*^th^ swine subject. The comparison of standard versus proposed methods was conducted based on the CoD, limits of agreement and RMNSE aggregated over all the swine subjects. Statistical significance was assessed using the repeated-measures ANOVA applied to CoD and RMSNE associated with standard versus proposed CO and TPR estimation methods. Difference was regarded as significant if *p* < 0.05.

## Results

The ranges of the physiological conditions associated with the experimental swine subjects are summarized in Table 1. It is obvious that all the subjects experienced large physiological changes due to medical interventions.

**Table 1:** **Physiological conditions of experimental swine subjects**.

**Subject ID**	**Mean HR [bpm]**	**Mean BP [mmHg]**	**Mean CO [lpm]**	**Mean TPR [mmHg/lpm]**
1	68/120 (89)	41.0/119.0 (59.7)	1.6/4.8 (2.8)	13.0/26.4 (21.3)
2	150/195 (177)	36.5/93.9 (66.0)	1.9/7.3 (4.1)	12.7/20.2 (16.1)
3	97/165 (120)	40.6/104.0 (71.6)	1.9/5.5 (3.8)	12.0/35.0 (18.8)
4	97/180 (120)	50.5/157.2 (78.6)	2.3/4.9 (3.1)	15.0/53.3 (25.4)
5	90/187 (125)	58.9/123.7 (88.4)	1.5/5.9 (3.8)	12.4/43.4 (23.3)
6	97/195 (120)	44.0/112.1 (79.7)	1.8/4.6 (3.0)	14.4/37.4 (26.6)
7	90/203 (136)	53.0/121.2 (87.7)	2.4/5.7 (3.7)	12.8/37.8 (23.7)
8	68/165 (129)	27.1/123.3 (80.4)	0.6/6.2 (3.9)	12.6/48.2 (20.6)
All	68/203 (123)	27.1/157.2 (79.3)	0.6/7.3 (3.4)	12.0/53.3 (22.9)

**Figure 2:**
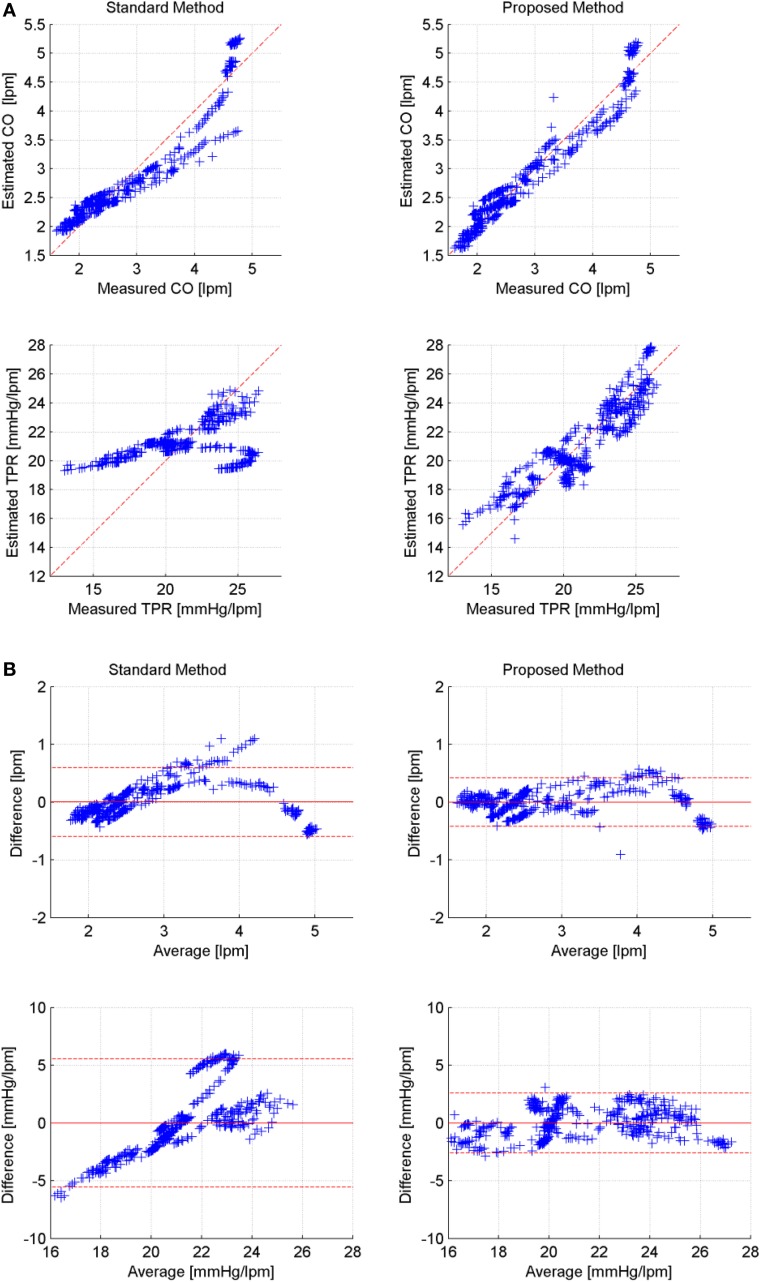
**Representative correlation and limits of agreement: measured versus estimated CO and TPR (Subject #1). (A)** Correlation between measured versus estimated CO and TPR (left: standard, right: proposed). **(B)** Bland-Altman plot between measured versus estimated CO and TPR (left: standard, right: proposed).

Table 2 lists CoD and RMSNE values associated with standard versus proposed CO and TPR estimation methods, where values associated with constant Equation (11) and pressure-dependent Equation (14) arterial compliance are presented for the proposed method. Table 3 presents the limits of agreement between measured versus estimated (using standard method and proposed method with pressure-dependent arterial compliance) CO and TPR. Figure 2 shows a representative (a) correlation between measured versus estimated CO and TPR and (b) Bland Altman plot between measured versus estimated CO and TPR, in which the proposed method is shown to outperform its standard counterpart. Aggregated over all animal subjects, the proposed method (both with constant and pressure-dependent arterial compliance) resulted in CoD and RMSNE values significantly different from those associated with the standard method (*p* < 0.05). The coefficients of the pressure-dependent arterial compliance model in Equation (13), determined by the calibration shown in Equation (17a), are shown in Table 4. On the average, arterial compliance was inversely proportional to mean BP (η_1_ < 0) but assumed positive values (η_2_ > 0), as physiologically anticipated.

**Table 2:** **CoD and RMSNE associated with standard versus proposed CO and TPR estimation methods**.

	**1**	**2**	**3**	**4**	**5**	**6**	**7**	**8**	**All**
**(A) CoD: CO ESTIMATION. VALUES IN () INDICATES IMPROVEMENT AGAINST STANDARD METHOD**									
Standard	0.895	0.985	0.823	0.663	0.712	0.600	0.720	0.876	0.737
Proposed (Constant C)	0.908	0.984	0.785	0.747	0.796	0.788	0.828	0.918	0.819 (11.1%)
Proposed ($C={\text{η}}_{1}\bar{P}+{\text{η}}_{2}$)	0.948	0.985	0.855	0.746	0.868	0.804	0.881	0.931	0.855 (16.0%)
**(B) RMSNE: CO ESTIMATION. VALUES IN () INDICATES IMPROVEMENT AGAINST STANDARD METHOD**									
Standard	9.9	5.4	11.8	9.1	17.5	16.0	12.7	22.4	13.7
Proposed (Constant C)	9.2	5.7	13.3	8.0	14.6	11.5	9.6	18.3	11.4 (16.8%)
Proposed ($C={\text{η}}_{1}\bar{P}+{\text{η}}_{2}$)	7.1	5.6	10.0	8.0	12.0	10.5	7.8	16.1	9.7 (29.2%)
**(C) CoD: TPR ESTIMATION. VALUES IN () INDICATES IMPROVEMENT AGAINST STANDARD METHOD**									
Standard	0.160	0.665	0.848	0.836	0.834	0.639	0.815	0.765	0.717
Proposed (Constant C)	0.647	0.716	0.888	0.905	0.668	0.765	0.872	0.692	0.781 (8.9%)
Proposed ($C={\text{η}}_{1}\bar{P}+{\text{η}}_{2}$)	0.782	0.711	0.872	0.907	0.758	0.789	0.899	0.748	0.808 (12.7%)
**(D) RMSNE: TPR ESTIMATION. VALUES IN () INDICATES IMPROVEMENT AGAINST STANDARD METHOD**									
Standard	14.2	6.0	12.5	10.3	10.6	14.1	11.0	12.6	11.8
Proposed (Constant C)	9.6	4.9	10.4	7.9	13.2	10.8	9.0	14.0	10.3 (12.7%)
Proposed ($C={\text{η}}_{1}\bar{P}+{\text{η}}_{2}$)	6.8	4.9	9.9	7.8	11.3	10.3	7.4	13.0	8.9 (24.3%)

**Table 3:** **Limits of agreement between measured versus estimated CO and TPR**.

	**1**	**2**	**3**	**4**	**5**	**6**	**7**	**8**	**All**
**(A) BLAND-ALTMAN STATISTICS: CO ESTIMATION (MEAN ± 1.96SD [mmHg])**									
Standard	0 ± 0.31	0 ± 0.15	0 ± 0.39	0 ± 0.29	0 ± 0.54	0 ± 0.43	0 ± 0.41	0 ± 0.47	0 ± 0.40
Proposed ($C={\text{η}}_{1}\bar{P}+{\text{η}}_{2}$)	0 ± 0.22	0 ± 0.15	0 ± 0.35	0 ± 0.25	0 ± 0.36	0 ± 0.30	0 ± 0.27	0 ± 0.35	0 ± 0.29
**(B) BLAND-ALTMAN STATISTICS: TPR ESTIMATION (MEAN ± 1.96SD [mmHg])**									
Standard	0 ± 2.84	0 ± 0.98	0 ± 2.43	0 ± 2.63	0 ± 2.71	0 ± 3.52	0 ± 2.83	0 ± 3.61	0 ± 2.85
Proposed ($C{\text{ = η}}_{\text{1}}\bar{P}{\text{ + η}}_{\text{2}}$)	0 ± 1.45	0 ± 0.91	0 ± 2.23	0 ± 1.99	0 ± 3.28	0 ± 2.69	0 ± 2.09	0 ± 3.73	0 ± 2.30

**Table 4:** **Model of pressure-dependent arterial compliance**.

	**1**	**2**	**3**	**4**	**5**	**6**	**7**	**8**	**All**
η_1_ × 10^5^	−10.6	6.7	−25.4	−0.74	−13.3	−2.6	8.2	−1.5	−4.9
η_2_ × 10^2^	4.2	2.4	6.1	2.2	4.2	3.6	2.1	3.2	3.5

## Discussion

### Validity and efficacy of proposed method

On the average, the proposed method resulted in 16% and 13% reduction in CoD for CO and TPR, respectively (see Tables 2A,C). It also resulted in 29% and 24% reduction of CO and TPR errors, respectively, if the pressure-dependent arterial compliance was used, and 17% and 12% reduction, respectively, if constant arterial compliance was used (see Tables 2B,D). In addition, the results with pressure-dependent arterial compliance were consistently superior to those with constant arterial compliance (see Table 2) with statistical significance. Table 3 indicates that the proposed method exhibits improved limits of agreement to the measured gold standard CO and TPR than its standard counterpart. Altogether, the above results suggest that (1) the fidelity of CO and TPR estimation may be improved by rigorous exploitation of multiple independent features in the BP waveform rather than resorting to a single feature (i.e., the pulse pressure) as in the case of the standard windkessel method, and (2) the explicit incorporation of pressure-dependent nature of arterial compliance may further benefit high-fidelity estimation of CO and TPR.

**Figure 3:**
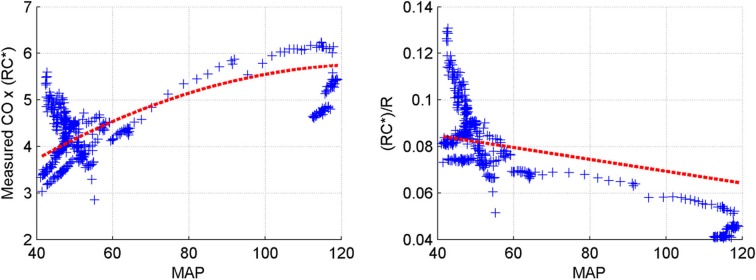
**Validity of linear pressure-dependent arterial compliance model (Subject #1)**.

Table 4 indicates that the identified models of arterial compliance exhibit physiologically meaningful behavior in most animal subjects (i.e., 6 out of 8), i.e., it is inversely proportional to the underlying BP (η_1_ < 0) and assumes positive values over the underlying BP values (η_2_ > 0). Figure 3 supports the validity of the pressure-dependent arterial compliance model used in this study: indeed, (14a) suggests that $RC^{*}\bar{Q}$ must depend on mean BP in a concave parabolic fashion (see the left panel of Figure 3):

(19a) $$RC^{*}\bar{Q}=\bar{P}\times ({\text{η}}_{1}\bar{P}+{\text{η}}_{2})={\text{η}}_{1}{\bar{P}}^{2}+{\text{η}}_{2}\bar{P},$$

while (14b) suggests that $\frac{RC^{*}}{R}$ must be linearly decreasing with respect to mean BP (see the right panel of Figure 3):

(19b) $$\frac{RC^{*}}{R}=C(P_{S},P_{D},\bar{P})={\text{η}}_{1}\bar{P}+{\text{η}}_{2}.$$

Figure 3 is consistent with what is anticipated, although some degree of deviation can be found quantitatively. In particular, the quantitative trend of arterial compliance shown in the right panel of Figure 3 is consistent with its typical pressure-dependent behavior reported in existing literature (e.g., Richter and Mittermayer, 1984). However, this was not the case for some animal subjects. In fact, although qualitatively similar observations to Figure 3 could be made for subjects #2 and #7, the trends were not as vivid and clear as those seen in Figure 3, which led to misleading outcomes for these subjects (see Table 4). This can be attributable to (1) the limited validity of the simple linear model of pressure-dependent arterial compliance (see Section “Limitations of the study” for more discussion) as well as to (2) the time-varying physiological conditions within the 8 s time window (in which case determining the optimal windkessel time constant RC^*^ associated with the time window can be challenging, since it is subject to change within the time window when the subject is affected by dynamic physiological states).

The validity of pressure-dependent arterial compliance (13) can further be assessed by scrutinizing the intercept coefficients η_3_ and *c*_2,*R*_. If the arterial compliance is truly dependent on mean BP in a linear fashion, (13) is fully valid and η_3_ and *c*_2,*R*_ must be zero. In the absence of any restrictions imposed on the intercepts, our regression analysis revealed that η_3_ and *c*_2,*R*_ assumed 0.93l pm and -10.6 mmHg/lpm on the average, respectively, suggesting that the proposed approach consistently underestimated CO and overestimated TPR. Considering the underlying CO and TPR values (see Table 1), the intercepts for both CO and TPR amounted to approximately 30% of the underlying values. However, noting that the slope coefficients in (17) were constrained at unity, it can be concluded that the proposed approach is able to estimate the absolute change of CO and TPR despite the non-negligible intercept coefficients. It is also important to point out that restricting *c*_2,*R*_ to zero in the calibration procedure (17) did not yield any noticeable degradation in performance of the proposed approach. Indeed, even when *c*_2,*R*_=0 was imposed (in which case η_3_ was also very close to zero), the proposed approach improved CoD of CO and TPR by 16.0% and 13.5%, respectively, and it also improved RMSNE of CO and TPR by 27.3% and 27.2%, respectively, when compared with its standard counterpart. In essence, the performance of the proposed approach was insensitive against whether or not *c*_2,*R*_ was restricted to zero. Therefore, it can be concluded that, though not perfect, (13) may be viewed as a valid approximation of arterial compliance that can be used with the proposed approach in order to reliably estimate CO and TPR.

### Mathematical analysis of proposed method

The advantage of the proposed windkessel method over the standard windkessel method in better estimating CO and TPR can be demonstrated by analyzing the systolic BP it represents as a function of *T*_*S*_. First, assuming *T*_*S*_ = 0, the systolic BP predicted in the proposed method in Equation (7a) becomes

(20) $$\begin{array}{c} \underset{T_{S}\to 0}{\lim}P_{S}=\underset{T_{S}\to 0}{\lim}\left\{P_{D}e^{-\frac{T_{S}}{RC}}+\bar{P}\frac{T}{T_{S}}\left[1-e^{-\frac{T_{S}}{RC}}\right]\right\}\\ =P_{D}+\bar{P}\frac{T}{RC}=P_{D}+\frac{\bar{Q}}{C}T, \end{array}$$

which is equivalent to Equation (3a). Thus, the proposed windkessel method reduces to its standard counterpart as expected, because the square wave approaches to impulse by shrinking *T*_*S*_ to zero. By virtue of its generalization capability, therefore, the proposed method can outperform its standard windkessel counterpart. On the other hand, if *T*_*S*_ = T, the systolic BP becomes

(21) $$\underset{T_{S}\to T}{\lim}P_{S}=P_{D}e^{-\frac{T}{RC}}+\bar{P}\left[1-e^{-\frac{T}{RC}}\right].$$

**Figure 4:**
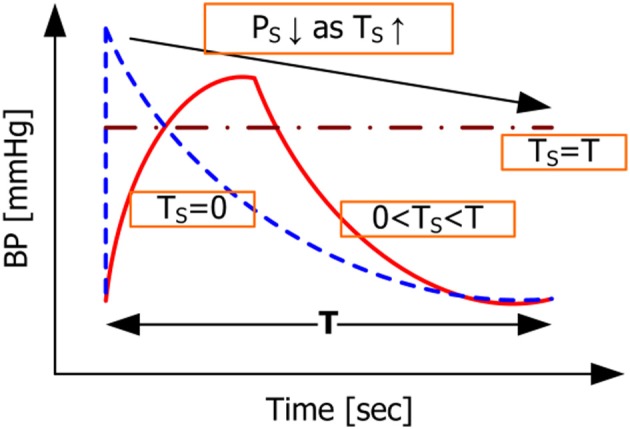
**Model-predicted BP waveforms associated with different values of *T*_*S*_**.

In the proposed method, an increase in *T*_*S*_ results in decrease in *T*_*D*_ since *T* is fixed from the measurement of heart period. Further, the relationship in Equation (7) on the ratio of systolic and diastolic BP given by $e^{-\frac{T_{D}}{RC}}$ together with their given measurements dictates that the ratio of *RC* and *T*_*D*_ must be kept at a constant regardless of the value of *T*_*D*_. Therefore, *RC* has to decrease as *T*_*S*_ increases in the proposed method. Since *T*_*S*_ ≈ *T* is equivalent to *T*_*D*_ ≈ 0, *RC* must be very small as well. If *RC* is sufficiently small such that $e^{-\frac{T}{RC}}\approx 0$ is valid, the systolic BP in Equation (21) can be approximated to:

(22) $$\underset{T_{S}\to T}{\lim}P_{S}=P_{D}e^{-\frac{T}{RC}}+\bar{P}\left[1-e^{-\frac{T}{RC}}\right]\approx \bar{P},$$

which is simply the behavior of the CVS in the steady state. Given the BP waveforms shown in Figure 4 associated with *T*_*S*_ = 0 and *T*_*S*_ = *T* as well as the constraint that the mean BP derived from the model should be equal to its measured counterpart regardless of the value of *T*_*S*_, it is obvious that the systolic BP in Equation (20) is greater than the one in Equation (22). Representative BP waveforms for 0 ≤ *T*_*S*_ ≤ *T* are also shown in Figure 4, where systolic BP is shown to decrease as *T*_*S*_ increases, which is anticipated from Equation (20) to Equation (22). Moreover, scrutinizing the mean BP constraint in Equation (9c) reveals that it essentially reduces to a constraint on diastolic BP weighted by *RC*. Indeed, it can be shown that evaluating the integration terms in Equation (9c) yields

(23) $$\tilde{\bar{P}}=\hat{\bar{P}}-\bar{P}=-\frac{RC}{T}({\hat{P}}_{D}-P_{D})=-\frac{RC}{T}{\tilde{P}}_{D}.$$

which essentially reduces Equation (10a) to the following:

(24) $$\left\{RC^{*},\;T_{S}^{*}\right\}=\arg\min[\left\|P_{S}-{\hat{P}}_{S}\right\|+\left(1+\frac{RC}{T}\right)\left\|P_{D}-{\hat{P}}_{D}\right\|].$$

Though yet to be fully validated, we expect that, compared with Equation (9b) which leads to the diastolic BP error term $\left\|P_{D}-{\hat{P}}_{D}\right\|$ in Equation (10a), (9c) which yields the mean BP error term $\left\|\bar{P}-\hat{\bar{P}}\right\|$ in Equation (10a) is useful in regularizing *RC* (i.e., keeping it from growing too large) as well as minimizing the diastolic BP error. Indeed, noting that systolic and diastolic BP can be tuned independently of each other with *T*_*S*_ and $\frac{T_{D}}{RC}$ in the proposed method, incorporating the mean BP error term into Equation (10a) via Equation (9c) allows the proposed method to exploit the range of {*RC*, *T*_*S*_, *T*_*D*_} beyond the standard windkessel method, i.e., the range corresponding to *T*_*s*_ > 0. In this context, a unique strength of the proposed method is its capability to characterize the CVS model by tuning the parameters {*RC*, *T*_*S*_, *T*_*D*_} in order to fit the model-predicted systolic, diastolic and mean BP to their measured counterparts, yielding a CVS model whose parameters can be utilized to improve the fidelity of CO and TPR estimation in comparison with the standard windkessel method that is solely built upon the pulse pressure.

### Limitations of the study

This study has a number of limitations. First, it was assumed that physiological condition of the animal subjects was time-invariant over each time window of 8 s. Although this should be a reasonable assumption for majority of the experimental data segments, it may not be well justified in part of the data, such as those corresponding to transient responses to the onset of drug administrations. Second, simple linear model was used to represent the pressure dependence of arterial compliance, although in reality it is known to be dependent on BP in highly nonlinear fashion. The linearity assumption may be valid for local approximation of arterial compliance within small pulse pressure range, but its validity will be deteriorated as the range of pulse pressure encompassed in the 8 s time window increases. In this regard, future work on the use of globally valid arterial compliance model in the proposed method is required. Third, despite the significantly large improvement in CO and TPR estimation provided by the proposed method, its utility may be limited to an extent by its requirement for calibration. In this regard, the hybrid use of proposed method with both constant and pressure-dependent arterial compliance can be a viable resolution. For example, CO and TPR can be estimated using Equation (11) until a number of CO measurements become available for calibration, after which Equation (14) can be used to estimate CO and TPR more accurately.

## Conclusion

In this paper, a novel universal approach was proposed to improve the performance of standard windkessel-model-based method in estimating the trend of CO and TPR. The validity and initial proof-of-principle of the proposed method was established via experimental evaluation and its comparison with the standard method. It has been suggested that the fidelity of CO and TPR estimation can be improved by rigorous exploitation of multiple features in the BP waveform to better characterize the CVS model. Future work is required in regards to further understanding on the capability and limitation of the proposed method as well as its application to extensions and variants of the standard windkessel-model-based CO and TPR estimation methods.

### Conflict of interest statement

The authors declare that the research was conducted in the absence of any commercial or financial relationships that could be construed as a potential conflict of interest.
